# Hyperuricemia in type 2 diabetic model KK-A^y^/Ta mice: a potent animal model with positive correlation between insulin resistance and plasma high uric acid levels

**DOI:** 10.1186/s13104-017-2897-x

**Published:** 2017-11-07

**Authors:** Shin-ichi Adachi, Fumiaki Yoshizawa, Kazumi Yagasaki

**Affiliations:** 1 Center for Bioscience Research and Education, Utsunomiya University, Mine-machi 350, Utsunomiya, Tochigi 321-8505 Japan; 2 Faculty of Agriculture, Utsunomiya University, Mine-machi 350, Utsunomiya, Tochigi 321-8505 Japan

**Keywords:** Hyperglycemia, Hyperuricemia, Insulin resistance, KK-A^y^/Ta mouse

## Abstract

**Objective:**

Hyperuricemia is recognized as a main cause of gout. Accumulating clinical evidence suggests that hyperuricemia is strongly associated with insulin resistance and abnormal glucose metabolism. However, there seem no proper animal models for investigating such associations. Ideal animal model is considered to be hyperuricemic as well as diabetic. Selecting the KK-A^y^/Ta mouse model, the relationship between hyperuricemia and insulin resistance has been studied to characterize such an animal model.

**Results:**

Male type 2 diabetic KK-A^y^/Ta and age-matched normal C57BL/6J mice were maintained on a basal 20% casein diet for 35 days. Food intake, body weight gain, levels of plasma uric acid, glucose, insulin, homeostasis model assessment of insulin resistance (HOMA-IR), and triglyceride in KK-A^y^/Ta mice were significantly higher than those in normal mice. Plasma uric acid levels showed significant positive correlations with plasma glucose, insulin, HOMA-IR and triglyceride levels. These results suggest that the KK-A^y^/Ta mouse strain is useful for studies on correlation between hyperuricemia and insulin resistance, and for those on effects of foods and their components on the relations.

## Introduction

Hyperuricemia is the high blood uric acid (UA) state resulting from overproduction of UA in the liver from purines synthesized in the body as well as those absorbed from the diet and/or reduced excretion of UA from the kidneys [1]. Hyperuricemia has been recognized as an important risk factor for the onset of gout [2, 3]. Hyperuricemia is also suggested to increase the risk of other diseases including hypertension, kidney disease and metabolic syndrome [4]. High dietary intake of purine-rich foods such as meats and sea foods increases UA levels in the blood [5]. Thus, we have contrived an assay system for UA production in vitro employing cultured AML12 hepatocytes and a hyperuricemic animal model in vivo induced by administration of purine bodies to ICR mice [6]. Employing these in vitro and in vivo systems, we have found that some food components such as quercetin [6] and taxifolin [7] inhibit UA production in the hepatocytes and successively confirmed that these polyphenols are capable of suppressing the purine bodies-induced hyperuricemia [6, 7] like allopurinol, a widely prescribed anti-hyperuricemic medicine, employed as a positive control agent in these studies. Since rodents including the mouse and rat have uricase which degrade uric acid to allantoin (humans lose uricase function), potassium oxonate (PO), a selectively competitive uricase inhibitor, is adopted to block the effect of hepatic uricase and to produce hyperuricemia in rodents [8]. PO-induced hyperuricemia in mice could serve as an animal model to evaluate the efficacy of drugs and possible therapeutic agents [8]. However, purine bodies- and PO-induced hyperuricemia seem to be acute and transient models and hence inappropriate for long-term observation on effects of candidate food components, despite that this acute model gives us the first information on their effectiveness in vivo.

In the meantime, we have been engaged in studies on antidiabetic effects of food components in type 2 diabetes (T2D) model animals such as KK-A^y^/Ta [9, 10], db/db [10–15] and ob/ob mice [16, 17]. Accumulating clinical evidence suggests that hyperuricemia is strongly associated with abnormal glucose metabolism and insulin resistance (IR) [18], and diabetic nephropathy [19]. Of these T2D model mice, KK-A^y^/Ta strain is well known as a possible model for human T2D nephropathy [20, 21]. So far, little is known about the relationship between hyperuricemia and IR in proper T2D model animals.

In this study, selecting KK-A^y^/Ta mice, we have investigated whether or not the mice would develop hyperuricemia and they show any relationships between blood UA levels and other parameters including IR.

## Main text

### Methods

Male diabetic KK-A^y^/Ta Jcl mice (4 weeks of age) and age-matched normal C57BL/6J Jcl mice were purchased from CLEA Japan, Inc., Tokyo, Japan. Normal and diabetic groups consisted of six mice per group, since six mice per each group showed appropriate sample size for statistical analyses of differences between two groups, i.e., normal and KK-A^y^/Ta groups [10]. They were housed in two groups in plastic cages and provided with tap water and pellet diet (CRF-1, Oriental Yeast Co., Tokyo, Japan) for 4 days ad libitum in a room with a temperature of 22 °C, a 12-h light and dark cycle (8:00–20:00 light phase), and they were thereafter individually housed in plastic cages and maintained on a basal 20% casein diet (20C, based on AIN-93G formula [22]: purified diets for laboratory rodents recommended by the American Institute of Nutrition) for another 8 days to acclimatize animals to the environment and semisynthetic diet. The composition of the 20C diet was as follows (dry weight basis): 20% casein (Oriental Yeast Co., Tokyo, Japan), 7% corn oil (Wako Pure Chemical Industries Ltd., Osaka, Japan), 13.2% α-cornstarch (Oriental Yeast Co.), 49.75% β-cornstarch (Oriental Yeast Co.), 3.5% mineral mixture (AIN-93G composition, Oriental Yeast Co.), 1% vitamin mixture (Oriental Yeast Co.), 0.25% choline bitartrate (Sigma-Aldrich Chemical Co., St. Louis, MO, USA), 0.3% l-cystine (Kyowa Hakko Kogyo Co., Ltd., Tokyo, Japan), and 5% cellulose powder (Oriental Yeast Co.). Food intake of each mouse was measured as follows: Each mouse was given the powdered 20C diet and water ad libitum in individual plastic cage. The 20C diet was put in a small glass pot (Shinano Seisakusho Co., Ltd., Cat# SN-950 No. 17A, Tokyo, Japan) that had an inner stainless plate accompanying small holes and a stainless lid. Using forelegs, a mouse ingested, through the small holes, the diet present underneath the plate. This plate with holes prevented that a mouse spilled the diet from its glass pot. The difference between the total weight of the pot including the diet, plate and lid between before and after feeding of a certain mouse was regarded as food intake of the mouse. Food intake was measured everyday.

**Fig. 1:**
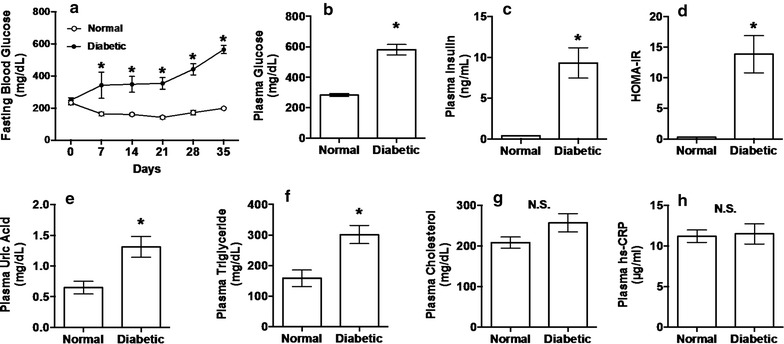
Changes of time-dependent fasting blood glucose (**a**), plasma glucose (**b**), insulin (**c**), HOMA-IR (**d**), uric acid (**e**), triglyceride (**f**), cholesterol (**g**) and hs-CRP (**h**) in normal C57BL/6J Jcl and diabetic KK-A^y^/Ta Jcl mice. Each value represents the mean ± SEM of 6 mice. *Significantly different from corresponding normal values at *P* < 0.05

**Fig. 2:**
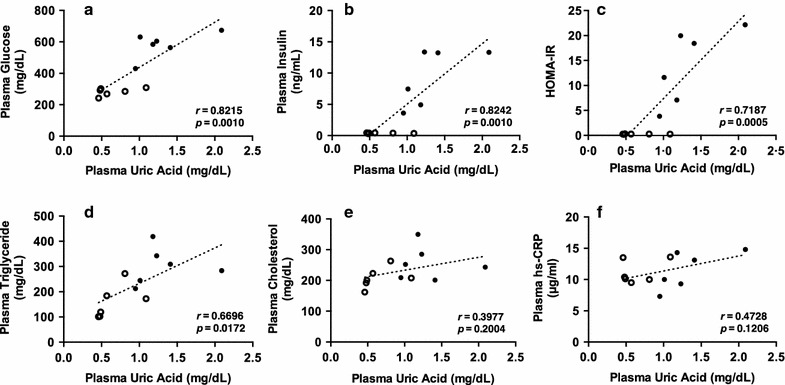
Correlations between plasma uric acid and other plasma parameters, glucose (**a**), insulin (**b**), HOMA-IR (**c**), triglyceride (**d**), cholesterol (**e**) and hs-CRP (**f**). Data were analyzed by Pearson correlation test in normal C57BL/6J and diabetic KK-A^y^/Ta mice. Symbol: ○, Normal; ●, Diabetic

After preliminary feeding for 12 (4 plus 8) days (day 0), mice were deprived of their diet at 9:00 but allowed free access to water until blood collection from tail vein 3 h later. Blood (10 μl) was burst in water (40 μl), 20% (w/v) trichloroacetic acid aqueous solution (50 μl) was added and test tubes containing the mixture were kept in ice-cold water. The mixture was then centrifuged and aliquots of the resultant supernatant was subjected to glucose determination with a commercial kit (Glucose CII Test Wako, Cat# 439-90901, Wako Pure Chemical Industries, Ltd., Osaka, Japan) and a microplate reader (SpectraMax M5, Molecular Devices, LLC, Sunnyvale, CA, USA) at 505 nm to measure fasting blood glucose (FBG) concentrations as described previously [11, 12]. Blood samples for FBG determination were collected every week (days 0, 7, 14, 21, 28 and 35). At the end of experimental period (day 35), the blood was collected after 6 h fasting under isoflurane anesthesia from the inferior vena cava into microtubes with heparin sodium. The blood samples were centrifuged at 5000×*g* for 10 min at 4 °C to obtain the plasma. The plasma was stored at − 80 °C until analyzed. Plasma glucose, insulin, UA, triglyceride, cholesterol and C-reactive protein (hs-CRP) were determined with commercial kits (Glucose CII Test Wako, Cat# 439-90901, Wako Pure Chemical Industries; Insulin assay kit, Cat# MS303, Morinaga Institute of Biological Science, Inc., Yokohama Japan; Uric Acid C-Test Wako, Cat# 437-17301, Wako Pure Chemical Industries; Triglyceride E-Test Wako, Cat# 432-40201, Wako Pure Chemical Industries; Cholesterol E-Test Wako, Cat# 439-17501, Wako Pure Chemical Industries; mouse High-sensitive CRP ELISA kit, Cat# KT-095, Kamiya Biochemical Co., Seattle, WA, USA). Homeostasis model assessment of insulin resistance (HOMA-IR) was calculated from glucose and insulin concentrations; HOMA-IR = fasting glucose level (mg/dl) × fasting insulin level (ng/ml)/405 [23]. Data are expressed as means ± SEM. Time-dependent changes of FBG concentrations (Fig. 1a) were analyzed by two-way repeated-measures ANOVA followed by Bonferroni’s multiple-comparisons test. Differences between two group means were compared by Student’s two-sided unpaired t test (Fig. 1b–h). Correlations between plasma UA and other plasma parameters were analyzed by Pearson correlation test (Fig. 2). *P* < 0.05 were considered statistically significant. These analyses were conducted by using the Prism 6 software package (GraphPad, San Diego, CA, USA).

### Results

Initial body weight (g/mouse), body weight gain (g/mouse/35 days) and food intake (g/mouse/35 days) of normal (n = 6) and diabetic (n = 6) mice were 21.1 ± 0.2 vs. 30.1 ± 0.6 (*P* < 0.0001), 5.5 ± 0.2 vs. 9.3 ± 0.6 (*P* < 0.001) and 179.9 ± 21.8 vs. 255.8 ± 9.7 (*P* < 0.01), respectively, indicating obesity and binge eating in KK-A^y^/Ta Jcl mice. The FBG levels of normal and diabetic mice were identical on day 0 and those of normal mice were almost constant during the experimental period of 35 days. In contrast, the FBG levels of diabetic mice gradually and significantly increased on day 7 and thereafter as compared with those of normal mice (Fig. 1a). Plasma glucose, insulin, HOMA-IR, UA and triglyceride levels were significantly higher in diabetic mice than in normal ones (Fig. 1b–f), whereas plasma cholesterol and hs-CRP levels were unchanged between two groups (Fig. 1g, h). Plasma UA levels showed significant positive correlations with plasma glucose, insulin, HOMA-IR and triglyceride levels, but did not show significant correlations with plasma cholesterol and hs-CRP levels (Fig. 2).

### Discussion

Allopurinol has been reported to prevent the progression of nephropathy and to reduce serum uric acid levels in KK-A^y^/Ta mice in comparison with those in allopurinol-untreated KK-A^y^/Ta mice [24], suggesting a possibility of the onset and involvement of hyperuricemia in KK-A^y^/Ta mice. However, the group of corresponding normal mice was not set up in the report. Allopurinol has been shown to also reduce serum uric acid levels even in the normal state [25]. Hence, it seems difficult to conclude from the report [24] that the KK-A^y^/Ta mouse strain induces hyperuricemia. Thus, we have intended to clarify this point in the present study. To our knowledge, we could confirm for the first time the definite onset of hyperuricemia in KK-A^y^/Ta mice as compared with its corresponding normal mice (Fig. 1e). Besides this finding, KK-A^y^/Ta mice are demonstrated to indicate a positive and significant correlation between plasma UA levels and IR. Thus, our finding of spontaneous hyperuricemia in KK-A^y^/Ta mice is considered to be useful for the long-term estimation of food components and natural resources against hyperuricemia, IR and diabetic nephropathy as well as hyperglycemia. In other T2D model mice, such as the db/db [26, 27] and ob/ob [28] strains, blood UA levels have been shown to significantly or slightly increase, but little is known about relationship between hyperuricemia and IR in these T2D mice so far. Very recently, knockout of the urate oxidase gene encoding uricase has been reported to provide a stable mouse model of hyperuricemia [29]. Uricase in the rodent liver degrades UA into allantoin, this forming an obstacle for establishing stable mouse models of hyperuricemia. Thus, functions of hepatic uricase gene and protein in KK-A^y^/Ta mice are required to be clarified in the near future.

In conclusion, the KK-A^y^/Ta mouse strain develops hyperuricemia and plasma UA levels positively and significantly correlate with other plasma parameters including triglyceride, glucose, insulin, and HOMA-IR 5 weeks after feeding of standard diet (20C). These results suggested that the KK-A^y^/Ta mouse strain is useful for studies on correlation between hyperuricemia and IR, and for those on effects of foods and their components on such relationships. However, further studies concerning time-dependent changes in parameters are required to learn when plasma UA levels commence to increase and how they correlate with other plasma parameters including glucose, insulin and HOMA-IR.

## Limitations

The limitation of this study includes lack of data on time-dependent changes in blood UA concentrations and small numbers of mice for correlation analyses. Therefore, it is difficult to clearly conclude that hyperuricemia in KK-A^y^/Ta mice is chronic and steady. Despite these limitations, plasma UA did increase 5 weeks after feeding and did show a positive and significant correlation between plasma UA and IR. These results suggest that the KK-A^y^/Ta mouse strain may be useful as a hyperuricemic model for long-term observation on effectiveness of candidate food components and natural resources.

## Abbreviations

hs-CRP: high-sensitive C-reactive protein
FBG: fasting blood glucose
HOMA-IR: homeostasis model assessment of insulin resistance
IR: insulin resistance
PO: potassium oxonate
T2D: type 2 diabetes
UA: uric acid
